# Supplementary material for: Understanding travel intention formation in government culture and tourism TikTok accounts: An integration of the SOR model and emotion appraisal theory
Source: PLoS One. 2026 May 18;21(5):e0349305. doi: 10.1371/journal.pone.0349305 (PMC13183215; doi:10.1371/journal.pone.0349305)
Supplement: S1 File — (PDF) [file pone.0349305.s001.pdf]

Questionnaire on the Influence of Government culture and tourism TikTok accounts on audience travel intentions

Dear Participants,

Thank you for taking part in this survey. This study examines audience experiences with government culture and tourism TikTok accounts. These accounts are officially certified and operated by government culture and tourism departments at different administrative levels and are typically identified by official verification badges on TikTok.

Your participation is voluntary and anonymous. All responses will be kept confidential and used solely for academic research purposes. If you are under 18 years old, participation requires permission from a parent or legal guardian. We sincerely appreciate your time and effort in completing this survey.

By clicking “Agree and Participate” and submitting the questionnaire, you confirm that you have read and understood the study information and consent to participate.

Thank you for your support!

I. Basic information.

1. Have you ever paid attention to government culture and tourism TikTok accounts or seen the short videos posted by them? (Single choice)

   ☐ I have watched the short videos and followed such accounts.

   ☐ I have watched the short videos but have not followed such accounts.

   ☐ I have not watched any short videos (selecting this option will end the survey)

2. What is your gender? (Single choice)

   ☐ Male ☐ Female

3. What is your age range? (Single choice)

   ☐ Before1980 ☐ Between 1980-1994 ☐ Between 1995-2009 ☐ After 2010

4. What is your highest degree (including studying) so far? (Single choice)

   ☐ High school degree/technical secondary school degree or below

   ☐ Junior college degree

   ☐ Undergraduate

   ☐ Master degree or above

5. What is your present occupation? (Single choice)

   ☐ School students ☐ Government organs and public institutions

   ☐ Enterprise personnel ☐ Self-employed or freelancers

   ☐ Farmer ☐ Retiree ☐ Other

6. What is your personal monthly disposable income? (Single choice)

   ☐Below 3000RMB ☐3001~5000RMB ☐5000~10000RMB ☐Above10000 RMB

7. How long do you use TikTok every day? (Single choice)

   ☐ Less than 30mins ☐ 30mins-1hour ☐ 1hour-2hours ☐ More than 2hours

II. Please rate the following statements based on your actual experience when watching short

videos published by government culture and tourism TikTok accounts or interacting with these accounts.

Responses are measured on a seven-point Likert scale.

1-Strongly disagree   2- Somewhat disagree   3-Disagree   4-Neutral

5- Somewhat agree   6-Agree   7- strongly agree

Variable	Measurement questions	1	2	3	4	5	6	7
**Information quality**	**Short videos posted by government culture and tourism TikTok accounts**							
	Of sufficient depth.							
	Specific.							
	Accurate.							
	Effective for planning a trip.							
	Useful for planning a trip.							
	Helpful for planning a trip.							
	Easy to understand.							
	Concise and appropriate.							
	Harmonious and consistent.							
**Service quality**	**I think government culture and tourism TikTok accounts**							
	Respond promptly to online comments.							
	Suggestions can receive timely feedback.							
	Have many interactive service features.							
	Trustworthy.							
	Sufficient professional knowledge.							
	Friendly and courteous.							
	Updated promptly and stably.							
	All kinds of services can be used.							
	Authoritative official information source.							
**Destination image perception**	**Based on government culture and tourism TikTok accounts, I perceive the destination as**							
	Attractive cultural or natural landscapes.							
	Unique environmental ambiance.							
	Favorable overall image.							
**Positive emotions**	**Viewing government culture and tourism TikTok accounts makes me feel**							
	Relaxed.							

	Pleasant.							
	Ecited.							
	Interesting.							
	Surprising.							
**Travel intentions**	**Based on my experience with government culture and tourism TikTok accounts, I intend to**							
	Recommend it to others.							
	Visit it in the future.							
	Say positive things about it to other people.							

Thank you once again for your support!
Research Team
