# Supplementary material for: Understanding travel intention formation in government culture and tourism TikTok accounts: An integration of the SOR model and emotion appraisal theory
Source: PLoS One. 2026 May 18;21(5):e0349305. doi: 10.1371/journal.pone.0349305 (PMC13183215; doi:10.1371/journal.pone.0349305)
Supplement: Attachment — Submitted filename: recommendation.doc [file pone.0349305.s003.doc]

**Overall recommendation: Major Revision**

This manuscript investigates how information quality and service quality of government Culture & Tourism Douyin accounts relate to audiences’ travel behavioral intentions, using the SOR framework and emotion appraisal theory with SEM on survey data (N=406). The topic is timely and relevant, and the empirical results are generally coherent. However, the paper currently reads as a largely “standard SEM application” and needs clearer theoretical contribution, stronger validity/robustness checks (especially for common method bias), and substantial language polishing. With major revisions, the manuscript may become suitable for publication.

**Major Comments**

**1) Clarify theoretical contribution beyond “applying SOR in a new context”**

The manuscript uses a well-established SOR structure (stimuli → organism → response). At present, the theoretical novelty is not sufficiently articulated. Please explicitly state what new theoretical insight emerges from studying government Douyin accounts rather than commercial/UGC contexts. Suggested actions:

Add a dedicated subsection (end of Introduction or start of Discussion) titled “Theoretical Contributions” with 2–3 specific points, e.g.:

how public-sector social media changes the role of service quality versus information quality.

why service quality effects are fully mediated in this context.

what this implies for extending SOR or public communication theory.

**2) Improve conceptual distinctiveness: information quality vs service quality**

On short-video platforms, boundaries between content features and interactive/service features can be blurred. Some service-quality items (e.g., “many interactive service features”) may overlap with perceptions of platform/content design. Please:

Provide a sharper conceptual rationale for treating information quality as content-based stimulus and service quality as interaction/response-based stimulus, and explain why these remain distinct in government TikTok  contexts.

Consider adding additional evidence of distinctiveness:

alternative discriminant validity checks (e.g., HTMT), or report cross-loadings / confirmatory factor comparisons.

3) **Common method bias: Harman’s single-factor test is insufficient**

The paper relies on Harman’s single-factor test, which is widely considered a weak diagnostic for CMB in self-reported cross-sectional designs. Please include at least one more rigorous approach, for example:

Common latent factor (CLF) technique in CFA/SEM and report whether substantive paths materially change, or Marker-variable approach, or Unmeasured latent method factor model.

Also clarify procedural remedies (e.g., anonymity, item order, instructions) beyond reporting statistics.

**4) Robustness and alternative models**

To strengthen confidence that the proposed mediation structure is not merely one plausible specification, please add at least one robustness/alternative-model test:

Compare the hypothesized model against reasonable alternative models, such as:

reversing organism variables (image → emotion vs emotion → image),

including a direct path from service quality to behavioral intentions but testing nested model comparisons, collapsing the two mediators into one (if justified) to show your model fits better.

Optional but valuable: multi-group SEM (e.g., Gen Z vs non-Gen Z, gender), given the demographic structure reported. Even if exploratory, it improves interpretability and external validity.

**5) Causal language should be toned down**

The dataset is cross-sectional and self-reported. Please avoid strong causal wording such as “influences/drive/enhances” unless you clearly frame them as associations. Suggested revisions:

Replace causal verbs with “is positively associated with / relates to / predicts (in the statistical sense)”.

Strengthen the limitations section explicitly: causal inference is limited and longitudinal/behavioral data are needed.

**6) Language and style require professional editing**

There are recurrent grammar issues, awkward phrasing, and some “Chinglish” expressions (e.g., “Government accounts have service attribute”). The manuscript would benefit from professional English editing. This is not cosmetic: readability affects reviewability and perceived rigor.

**Minor Comments**

Hypotheses numbering/logic: H1 and H3 appear overlapping (information quality → behavioral intentions). Please check redundancy and ensure each hypothesis is unique and consistent with the model narrative.

Measurement detail: provide the full item wording in a supplement (or an appendix) and specify how items were adapted (what modifications were made). This improves reproducibility.

Model fit reporting: consider reporting additional indices commonly requested (e.g., SRMR) and note thresholds with citations.

**Summary**

The manuscript addresses a relevant topic and reports plausible findings, but it requires major revisions to strengthen theoretical contribution, ensure construct distinctiveness, and substantially polish English writing.
