# Supplementary material for: Understanding travel intention formation in government culture and tourism TikTok accounts: An integration of the SOR model and emotion appraisal theory
Source: PLoS One. 2026 May 18;21(5):e0349305. doi: 10.1371/journal.pone.0349305 (PMC13183215; doi:10.1371/journal.pone.0349305)
Supplement: Attachment — Submitted filename: Response to Reviewers.doc [file pone.0349305.s006.doc]

Response Letter

Dear editors and reviewers,

We are very grateful for your constructive comments and suggestions for our manuscript entitled "Investigating the effects of information quality and service quality of government culture and tourism TikTok accounts on audience travel behavioral intentions" (ID:PONE-D-25-66483). Your comments are very valuable and helpful for improving our manuscript. In the following, we provide point-by-point responses to all comments.

We have carefully revised the manuscript to ensure that all changes are clearly presented, and we hope that the revised manuscript can meets the requirements for publication. The main revisions in the new manuscript are:

1. In the introduction, we added a review of the SOR model and emotion appraisal theory to construct the theoretical framework for this study. The introduction also clearly outlines the theoretical contributions of the study.
2. In the literature review and hypothesis development sections, we reorganized existing studies and proposed the hypotheses based on a critical review of prior research. To maintain clarity, unrelated hypotheses were removed, keeping only the direct effects, parallel mediation effects, and sequential mediation effects between information quality, service quality, and audience travel intentions.
3. In the variable measurement section, We added a comparison between original and adapted measurement items, using selected examples to demonstrate how the scales were adjusted to fit the research context.
4. In the sample collection section, we provided additional details about the survey procedures, including the sampling frame, recruitment channels, and measures to prevent duplicate or careless responses. In accordance with standard academic practice, an ethics statement has been incorporated into the manuscript.
5. In the data analysis section, we conducted confirmatory factor analysis with competing models to assess construct validity. In addition, we incorporated an unmeasured latent method factor into the structural model into the structural model to assess the potential impact of common method bias.
6. We have substantially revised the Discussion to strengthen the theoretical interpretation. The revision integrates the heuristic–systematic model and emotion appraisal theory, clarifies the distinct roles of information quality and service quality, and shows that destination image perception occurs before and is linked to positive emotions, highlighting how cognitive evaluation is linked to emotional responses and strengthening the study’s theoretical insights.
7. The limitations section has been revised to acknowledge the cross-sectional design, context dependence, and linear modeling assumptions, and we suggest that future research explore longitudinal, cross-platform, and nonlinear approaches to better understand user responses in social media environments.

We would also like to note that, following the substantial revisions made to strengthen the theoretical framework and clarify the study’s contributions, we have revised the manuscript title to better reflect the updated focus and theoretical positioning of the study.**The revised title is:** **“Understanding travel intention formation in government culture and tourism TikTok accounts: An integration of the SOR model and emotion appraisal theory”.** The change concerns wording only and does not affect the study’s objectives, data, or findings. We hope that the revised title improves the clarity and accuracy of the manuscript’s presentation.

We sincerely appreciate the constructive feedback from the editors and reviewers. The insightful comments have significantly improved the clarity, rigor, and theoretical contribution of our manuscript. We hope that the revisions have adequately addressed the concerns raised and that the manuscript is now suitable for publication in PLOS ONE.

Best regards,

Yuan Sun (on behalf of all co-authors)

**Response to Reviewer 1:**

**Comment1：**

The introduction does not sufficiently highlight the theoretical innovation of the study. While the introduction outlines the operational complexity of government accounts and mentions the dual attributes of policy advocacy and public service, it fails to clearly articulate how this research advances existing theoretical frameworks. For instance, it states that government accounts must "balance policy advocacy and public service responsibilities," but does not explicitly explain how integrating service quality into the SOR model—typically focused on information or system quality—creates a novel "dual-driver" framework for public sector communication. The concluding paragraph of the introduction should be revised to sharply contrast this study's integrated model (information + service quality) with prior SOR applications in commercial or UGC contexts, thereby underscoring its distinct theoretical contribution to understanding government social media effects.

We sincerely appreciate you for the insightful and constructive comments. Your suggestions provided valuable guidance for refining the theoretical positioning and rigor of the manuscript, and we have carefully revised the relevant sections accordingly.

**Response1：**

In response to your suggestions, we have systematically revised this section and further clarified the theoretical contributions and incremental value of our study in relation to existing research.

First, in the opening paragraph of the introduction, we elaborated on the composite functional characteristics of government culture and tourism TikTok accounts. We explicitly emphasize that such accounts not only serve as channels for tourism information dissemination and destination image construction, but also fulfill responsibilities related to public communication and online service responsiveness. This dual role reflects their institutional attributes and public responsibilities, distinguishing them from commercial or user-generated content (UGC) accounts.

Second, in the subsequent paragraph, we expanded the literature review by incorporating studies from both tourism short-video research and the e-government domain. We point out that prior studies have either focused on information content or platform-related technological attributes, or concentrated on administrative service-oriented e-government contexts. Relatively little attention has been paid to government social media accounts operated by culture and tourism departments, which simultaneously perform functions of “image promotion” and “public engagement.” Therefore, within this hybrid communication environment that integrates information dissemination and public engagement, the mechanisms through which service quality and information quality are associated with audience travel intentions remain insufficiently explored.

Third, at the end of the introduction, we added a concise statement highlighting the theoretical contributions of this study in three aspects. Initially, we extend the stimulus variables in the context of government social media tourism marketing by incorporating service quality as a relational factor into the analytical framework. Furthermore, we systematically explain how information quality and service quality are related to audience behavioral responses through distinct relational pathways, thereby constructing a more comprehensive dual-driving mechanism. Finally, at the organism level, we refine the processes of cognitive appraisal and emotional processing. This provides a clearer view of the internal psychological mechanisms. These processes explain how stimuli are associated with behavioral responses.

**Comment2：**

The literature review lacks critical synthesis and reads more like a summary list, failing to establish a clear dialogue with existing research. The review categorizes short-video marketing into three paradigms but does not critically analyze their limitations when applied to government-operated accounts. For example, it notes that most studies focus on information quality while overlooking service quality but does not engage with relevant public administration or e-government literature discussing the service role of official social media (e.g., Zhang et al., 2017 on government WeChat service quality). To strengthen the argument, the review should explicitly position itself against prior work, explaining why the service dimension is especially salient and under-theorized for government tourism accounts, and how this study's combined focus addresses a defined gap.

**Response2：**

Following your suggestion, we have substantially revised this section to strengthen the theoretical dialogue with existing studies.

First, in the second paragraph of the introduction, we restructured the review of tourism short video marketing studies. Through a systematic examination of prior research, we point out that most studies on social media tourism marketing primarily focus on stimulus factors such as information content characteristics, contextual cues, or platform-related technological attributes, while paying relatively limited attention to service quality as a relational dimension. At the same time, we analyzed relevant studies from the e-government field. Although e-government research emphasizes service-related elements such as reliability, responsiveness, and perceived trust, its core function is centered on administrative service delivery and efficiency improvement. This functional orientation differs fundamentally from the logic of government social media tourism marketing, which emphasizes image construction, emotional communication, and public engagement. Therefore, existing e-government service quality frameworks may not be directly applicable to the context of government culture and tourism social media marketing. Based on this analysis, government culture and tourism TikTok accounts serve both image promotion and public communication functions. In this dual context, the service dimension has both institutional and interactive significance, but remains insufficiently theorized. This gap constitutes a key entry point for our study.

Second, in the third paragraph of the introduction, we added an integrated discussion of the SOR model and the emotion appraisal theory. This addition provides a theoretical justification for incorporating both information quality and service quality into a unified analytical framework. On this basis, we propose a dual-path mechanism model to address the identified theoretical gap.

Furthermore, in the hypothesis development section, we refined and reorganized the relevant literature. By systematically reviewing prior findings and explicitly identifying their limitations, we proposed our research hypotheses in a more logically and critically synthesized manner.

**Comment3：**

The methods section is described too briefly, omitting key details necessary for replicability and assessment of validity. The adaptation process of measurement scales is only vaguely described as involving "translation and back-translation" and expert review. The manuscript should provide specific examples of how items were modified to fit the context of government Douyin accounts (e.g., original scale item vs. adapted item). Furthermore, the sampling procedure states that questionnaires were distributed "via WeChat and Douyin" but lacks details on the sampling frame, recruitment channels, or measures to prevent duplicate or inattentive responses (e.g., attention checks). Additionally, while Harman's single-factor test is mentioned, the result (38.93%) is not reported in the main text or tables. These omissions hinder the evaluation of the study's rigor.

**Response3：**

Following your suggestion, we have supplemented the Method section to provide clearer information on sampling procedures, measurement adaptation, and validity assessments, thereby enhancing the reproducibility and rigor of the research.

First, regarding data collection procedures, we have added detailed instructions on recruitment channels, screening criteria, and data quality control measures. To ensure that respondents had relevant experiential backgrounds, we provided a brief description of “government culture and tourism TikTok accounts” on the first page of the questionnaire and explicitly required that participants have prior experience viewing such short videos. The survey was administered through the Wenjuanxing platform, which was configured to prevent multiple submissions by blocking duplicate responses from the same login account, IP address, and device. Based on testing about response time, questionnaires completed in less than 90 seconds were considered invalid and removed from the dataset to minimize careless responses. In terms of recruitment channels, on WeChat the questionnaire was mainly distributed through tourism-interest groups and shared through appropriate snowball diffusion; on TikTok, we used private messages to invite participation, randomly selecting users who had commented on short videos posted by government culture and tourism accounts. These measures helped ensure better alignment between the sample and the research context.

Second, regarding common method bias, we implemented more rigorous statistical tests. In addition to reporting the Harman’s single-factor test result (with the first factor explaining 38.93% of the variance), we further introduced an unmeasured latent method factor into the baseline structural model to construct an extended model (Model M7). According to the criteria proposed by Chen (2007), changes in fit indices below 0.02 indicate no substantial improvement in model fit. Therefore, the inclusion of the unmeasured latent method factor did not significantly enhance model fit, suggesting that common method bias is unlikely to pose a serious threat to the findings. These results have now been clearly reported in the manuscript.

Third, we have added a comparison table in the Appendix comparing the original measurement scales with the adapted items.

**Comment4：**

4. The discussion of results is relatively superficial, primarily restating findings without deeply interpreting their theoretical significance or contextualizing them within the broader literature. For instance, the key finding that service quality exerts only an indirect effect (full mediation) while information quality has both direct and indirect effects is noted but not sufficiently explained. The discussion attributes this to "fragmented browsing" habits but does not connect it to theoretical mechanisms such as the heuristic processing of authoritative information versus the systematic evaluation required for service interactions. Moreover, the contrast with commercial tourism accounts—where service quality may have a direct impact—is hinted at but not substantiated with references to comparable studies. The discussion should explicitly link each major result back to the SOR and emotion appraisal frameworks, explore alternative explanations, and compare findings with related work to clarify the unique behavioral pathways in government social media contexts.

**Response4：**

Following your suggestion, we have substantially revised and expanded the discussion, with improvements made in the following aspects:

First, regarding the key finding that information quality exerts both direct and indirect effects, whereas service quality demonstrates only a fully mediated effect, we moved beyond the prior contextual explanation of “fragmented browsing habits.” Following your suggestion, we introduced the Heuristic–Systematic Model (HSM) as a complementary theoretical perspective to explain the underlying information-processing mechanisms. Specifically, information released by government culture and tourism accounts carries strong authority and official endorsement, which may trigger heuristic processing and thus directly predict travel intentions. In contrast, service quality involves perceptions of interaction, responsiveness, and relational evaluation, which require more systematic processing. Therefore, it is more likely to be indirectly associated with audience travel intentions through cognitive and emotional mediating mechanisms. This theoretical extension helps explain the structural differences in the action paths of the two types of stimulus variables.

Second, we strengthened dialogue and comparison with prior studies. In the revised manuscript, all major empirical findings are discussed in relation to relevant literature. For example, the mediating roles of destination image perception and positive emotion between information quality, service quality, and behavioral intention are consistent with previous studies. The results indicate that this psychological process also applies to government culture and tourism social media marketing contexts.

Third, we deepened the theoretical discussion of the internal structure within the organism level. Unlike some prior studies that propose an emotion–image pathway, our findings suggest a pattern in which destination image perception is positively associated with emotional experience, indicating a cognitive–emotional ordering. This result aligns with the core assumptions of the emotion appraisal theory and enriches the explanatory power of the SOR framework regarding the internal mechanisms of the organism component in social media tourism marketing contexts.

We sincerely appreciate your valuable comments and suggestions. We hope that the revisions and explanations provided have adequately addressed your concerns and improved the overall quality of the manuscript.

**Response to Reviewer 2:**

**Comment1：**

Clarify theoretical contribution beyond “applying SOR in a new context”
The manuscript uses a well-established SOR structure (stimuli → organism → response). At present, the theoretical novelty is not sufficiently articulated. Please explicitly state what new theoretical insight emerges from studying government Douyin accounts rather than commercial/UGC contexts. Suggested actions:
Add a dedicated subsection (end of Introduction or start of Discussion) titled “Theoretical Contributions” with 2–3 specific points, e.g.:
how public-sector social media changes the role of service quality versus information quality.
why service quality effects are fully mediated in this context.
what this implies for extending SOR or public communication theory.

**Response1：**

We sincerely thank you for your insightful and constructive suggestions. Following your recommendations, we have revised the manuscript accordingly and added a new paragraph at the end of the Introduction to clearly articulate the theoretical contributions of this study.

First, this study clarifies how information quality and service quality operate through different pathways in the government social media marketing context. Most prior studies, grounded in commercial or UGC settings, generally assume that both information quality and service quality directly predict travel intentions. However, our findings show that in the context of government culture and tourism TikTok accounts, the two stimulus variables follow structurally different pathways. Information quality is directly associated with behavioral intention and also exerts indirect effects through psychological mediators, whereas service quality operates only through full mediation. This result suggests that the governmental context leads stimulus variables to follow different association pathways with audience travel intentions, thereby enriching our understanding of behavioral formation mechanisms in government communication settings.

Second, we provide a theoretical explanation for the fully mediated mechanism of service quality. Unlike traditional service marketing theory, which typically assumes a direct positive relationship between service quality and behavioral intention, our findings indicate that in the GCTTA context, service quality does not directly influence behavioral intention. Instead, it is associated with behavioral intention through destination image perception and positive emotion. We interpret this result from the perspectives of public trust, authority signaling, and information-processing mechanisms. In government-led tourism communication, service quality is more likely to be perceived as a signal of authority, professionalism, and responsiveness rather than as an immediate persuasive driver. Its influence operates through systematic processing by enhancing cognitive evaluation and emotional experience, which are subsequently linked to behavioral responses. This finding provides a new perspective for distinguishing between transaction-oriented service quality and public trust-oriented service quality.

Third, this study advances the structural development of the SOR framework in government communication contexts. By integrating the emotion appraisal theory, we decompose the organism component into cognitive evaluation (destination image perception) and emotional experience (positive emotion), and provide empirical evidence for their sequential relationship. The findings support a cognition-to-emotion progression, thereby extending the traditional stimulus–emotion–response structure into a stimulus–cognition–emotion–response framework. This refinement deepens the explanation of internal psychological processes within the SOR model and enhances its applicability in government social media tourism marketing research.

**Comment2：**

2) Improve conceptual distinctiveness: information quality vs service quality
   On short-video platforms, boundaries between content features and interactive/service features can be blurred. Some service-quality items (e.g., “many interactive service features”) may overlap with perceptions of platform/content design. Please:
   Provide a sharper conceptual rationale for treating information quality as content-based stimulus and service quality as interaction/response-based stimulus, and explain why these remain distinct in government TikTok  contexts.
   Consider adding additional evidence of distinctiveness:
   alternative discriminant validity checks (e.g., HTMT), or report cross-loadings / confirmatory factor comparisons.

**Response2：**

In response to your suggestions, we have revised the manuscript in the following ways:

First, we have further clarified the conceptual distinction between information quality and service quality in the hypothesis development section. Information quality is defined as the intrinsic quality characteristics of short video, including accuracy, completeness, and usefulness. Its primary function is to help audiences understand destination attributes and reduce decision uncertainty; therefore, it can be conceptualized as a content-oriented cognitive stimulus. In contrast, service quality emphasizes the supportive performance of government TikTok accounts in interaction and communication, such as responsiveness, engagement, and problem-solving ability. It focuses on how account operators interact with the audience and thus represents an interaction-oriented relational stimulus. This conceptual differentiation not only strengthens the theoretical clarity of the constructs but also provides a foundation for explaining their distinct structure.

Second, we further assessed discriminant validity at the empirical level. We conducted a competing confirmatory factor analysis (CFA) by comparing the proposed five-factor measurement model with an alternative four-factor model that combined information quality and service quality into a single construct. The results show that the five-factor model provides a better fit than the combined model, supporting the structural distinction between information quality and service quality. This finding offers statistical evidence for treating the two constructs as separate stimulus variables within the theoretical framework.

Through these revisions, we clarify the structural differentiation between the two stimulus variables in the public-sector social media context and strengthen both the conceptual and empirical foundations for subsequent discussions of their distinct effects.

**Comment3：**

Common method bias: Harman’s single-factor test is insufficient
The paper relies on Harman’s single-factor test, which is widely considered a weak diagnostic for CMB in self-reported cross-sectional designs. Please include at least one more rigorous approach, for example:
Common latent factor (CLF) technique in CFA/SEM and report whether substantive paths materially change, or Marker-variable approach, or Unmeasured latent method factor model.
Also clarify procedural remedies (e.g., anonymity, item order, instructions) beyond reporting statistics.

**Response3：**

We agree that relying solely on Harman’s single-factor test is insufficient to rigorously diagnose potential method bias in self-reported cross-sectional data. In response to your suggestion, we have strengthened the manuscript by addressing CMB from both procedural and statistical perspectives.

First, we clarified and enhanced the procedural remedies implemented during data collection.

1) The questionnaire was administered anonymously to reduce social desirability bias.
2) We expanded the description of the sampling procedure, including recruitment channels, screening criteria, and data quality control measures. To ensure respondent relevance, the homepage of the questionnaire provided a clear explanation of government culture and tourism TikTok accounts, and only individuals with prior viewing experience were eligible to participate.
3) Technical restrictions were implemented through the survey platform (Wenjuanxing), including preventing multiple submissions from the same login account, IP address, or device.
4) Based on pre-testing, responses completed in less than 90 seconds were considered invalid and removed to minimize careless or inattentive responses.
5) Regarding recruitment channels, the survey was distributed in tourism-interest groups on WeChat and further disseminated through controlled sharing, while on TikTok, users who had previously commented on government culture and tourism short videos were randomly selected and invited via private messages. These procedures helped reduce potential homogeneity and systematic bias in the sample.

Second, we further examined the potential influence of common method bias on the structural model. An unmeasured latent method factor was added to the baseline model (M1) alongside the five theoretical constructs—information quality, service quality, positive emotion, destination image perception, and behavioral intention—resulting in Model M3.Then we compared the model fit indices of M1 and M7. The changes in fit indices were minimal (ΔGFI = 0.015, ΔRMSEA = 0.003, ΔNFI = 0.012, ΔIFI = 0.009, and ΔCFI = 0.009). According to the criterion proposed by Chen (2007), changes smaller than 0.02 indicate no meaningful improvement in model fit. Therefore, adding the unmeasured latent method factor did not substantially improve model fit, suggesting that common method bias is unlikely to threaten the validity of the findings.

These additional procedural and statistical tests further enhance the methodological rigor and the robustness of the findings.

**Comment4：**

3) Robustness and alternative models
   To strengthen confidence that the proposed mediation structure is not merely one plausible specification, please add at least one robustness/alternative-model test:
   Compare the hypothesized model against reasonable alternative models, such as:
   reversing organism variables (image → emotion vs emotion → image),
   including a direct path from service quality to behavioral intentions but testing nested model comparisons, collapsing the two mediators into one (if justified) to show your model fits better.
   Optional but valuable: multi-group SEM (e.g., Gen Z vs non-Gen Z, gender), given the demographic structure reported. Even if exploratory, it improves interpretability and external validity. We believe the result demonstrates the robustness of the proposed mediation structure.

**Response4：**

Following your suggestion, we conducted an additional robustness test of the proposed model. An alternative measurement model (M3) was constructed, in which destination image perception and positive emotion were combined into a single organism construct. The detailed results are presented in Table 3 of the revised manuscript. Compared with the hypothesized five-factor model (M1), the alternative model showed substantially poorer model fit. This result suggests that combining the two mediating variables weakens the model’s explanatory structure. The findings support the conceptual distinction between cognitive and emotional organism states and justify modeling them as separate mediators in the proposed framework. The corresponding analyses and explanations have been added to the revised manuscript to further strengthen the robustness of the model specification.

**Comment5：**

5) Causal language should be toned down

The dataset is cross-sectional and self-reported. Please avoid strong causal wording such as “influences/drive/enhances” unless you clearly frame them as associations. Suggested revisions:

Replace causal verbs with “is positively associated with / relates to / predicts (in the statistical sense)”.

Strengthen the limitations section explicitly: causal inference is limited and longitudinal/behavioral data are needed.

**Response5：**

Following your recommendation, we conducted a thorough review of the entire manuscript. We revised potentially causal expressions such as “affect,” “influence,” “drive,” and “enhance,” replacing them with more cautious, association-based wording, such as “is positively associated with,” “is related to,” “is linked to,” and “predicts.” For example, all instances of "influence" in the hypotheses have been revised to "is positively associated with."

In addition, we have revised the limitations section to explicitly acknowledge that the cross-sectional design limits our ability to draw strong causal inferences. We emphasize that future research using longitudinal designs or behavioral data would be needed to establish causal relationships more rigorously.

**Comment6：**

Language and style require professional editing
There are recurrent grammar issues, awkward phrasing, and some “Chinglish” expressions (e.g., “Government accounts have service attribute”). The manuscript would benefit from professional English editing. This is not cosmetic: readability affects reviewability and perceived rigor.

**Response6：**
We acknowledge that the language and writing style of the original manuscript affected its overall readability. Following your suggestion, we conducted a thorough revision of the entire manuscript. The revision focused on improving grammatical accuracy, clarity of expression, and academic writing standards. We carefully corrected grammatical errors, awkward phrasing, and “Chinglish” expressions throughout the paper.Although we have made our best effort to refine the language, some imperfections may still remain. We sincerely welcome any further guidance.

**Comment7：**

Minor Comments

Hypotheses numbering/logic: H1 and H3 appear overlapping (information quality → behavioral intentions). Please check redundancy and ensure each hypothesis is unique and consistent with the model narrative.

**Response7：**

We sincerely thank the reviewer for the careful review. Following your suggestion, we have reorganized and refined the hypothesis structure based on theoretical logic. We retained the direct effects, parallel mediation effects, and sequential mediation effects, resulting in a total of 8 hypotheses, while removing redundant or unrelated hypotheses. These revisions make the conceptual model clearer and more rigorous.

**Comment8：**

Measurement detail: provide the full item wording in a supplement (or an appendix) and specify how items were adapted (what modifications were made). This improves reproducibility.

Model fit reporting: consider reporting additional indices commonly requested (e.g., SRMR) and note thresholds with citations.

**Response8：**

Following your suggestion, we have further improved the reporting of measurement details and model fit information in the revised manuscript.

First, we have now provided the survey questionnaire and dataset used for analysis in supplementary files to enhance the reproducibility of this study. Additionally, to clarify how the items were contextualized for our research context, we have added a detailed explanation of some items in the Variable measurement section (3.1). Specifically, we now describe the adaptation process with illustrative examples. For example, "The original item assessing whether the information provided by an official WeChat account is authoritative and trustworthy, proposed by Zhang (2017), was adapted to assess whether government cultural and tourism TikTok accounts (GCTTAs) serve as authoritative official sources of information. This adaptation improves contextual relevance and clarity of measurement while preserving the original construct meaning."

Second, regarding model fit reporting, we have added the SRMR index to the model evaluation results. In addition, the recommended threshold values for mode fit indices have been explicitly reported in the manuscript to support our assessment of model fit.

We sincerely appreciate your valuable comments and suggestions. Through these revisions, we have strengthened the theoretical positioning of the study, incorporated additional robustness and validity checks, and improved the overall clarity and rigor of the manuscript. We hope that the revisions have adequately addressed your concerns and enhanced the overall quality of the manuscript.

**Response to Reviewer 3:**

**Comments on the introduction：**

1. For publication in an international journal, the use of proper nouns must be precise. The authors should verify whether "Douyin" is the standard accepted English terminology in current academic literature, or if it requires clarification (e.g., explicitly defining it as the Chinese version of TikTok) to ensure clarity for an international readership.
2. The authors assert that “the operational logic of government culture and tourism accounts is more complex” due to the need to balance policy advocacy and public service with user engagement. While this distinction is intuitive and pivotal to the study, the statement is currently presented without sufficient theoretical grounding.
3. The authors postulate that government culture and tourism accounts face a complex dual mandate involving 'policy advocacy.' However, this claim must be aligned with empirical reality. Based on the examples provided in the manuscript, the content appears to function primarily as generic destination promotion, indistinguishable from standard user-generated content. The authors need to clarify whether policy advocacy is genuinely embedded in the analyzed videos. If these accounts merely replicate the promotional style of the general public, the authors must explicitly delineate what constitutes the unique difference between government and public accounts to justify the theoretical necessity of focusing specifically on this group.
4. There is a noticeable lack of logical cohesion between the second and third paragraphs of the Introduction.
5. The study adopts the S-O-R framework and Appraisal Theory of Emotion as its theoretical foundation. However, the rationale for selecting these specific lenses remains unclear. The authors have not sufficiently explained why these theories are the most appropriate tools for this specific research context, nor have they articulated their unique value in explaining the observed phenomena.
6. The Introduction currently lacks a deep exploration of the study's theoretical and practical implications. The authors should elaborate on these aspects to clearly demonstrate how the research advances existing theoretical boundaries and provides substantive, actionable insights for destination management, rather than relying on broad generalizations.

**Response：**

We sincerely thank you for the insightful and detailed comments. Your suggestions helped us standardize terminology for an international readership, clarify the theoretical and practical justification for focusing on government culture and tourism accounts, and strengthen the rationale for adopting and integrating the SOR framework with emotion appraisal theory. The detailed revisions are provided below.

1. Thank you for pointing out the terminology issue. Upon reviewing the existing literature, we found that studies on the Douyin platform often refer to it as "TikTok." Although this study focuses on the Chinese version (Douyin), we have used the term "TikTok" throughout the manuscript to align with international literature. We have clarified this in the revised text.
2. We have added a clarification in the revised manuscript regarding the operational characteristics of government culture and tourism TikTok accounts. Specifically, we clarify that these accounts serve dual roles in digital media environments: disseminating information and facilitating public communication. In addition to destination promotion, account operators actively engage with users, such as responding to comments, providing additional activity information, and addressing complaints and suggestions. These practices reflect the public service attributes that distinguish government accounts from general commercial or user-generated accounts. Through these revisions, we provide a clearer explanation for the “operational complexity” of government culture and tourism TikTok accounts from the perspectives of role positioning and functional attributes.
3. We carefully re-examined the content of the government culture and tourism TikTok accounts. Our review indicates that although some videos do include promotional information related to preferential policies, such content represents only a relatively small proportion of the overall sample. In light of this reality, we have revised the manuscript to avoid overemphasizing “policy advocacy” as a dominant feature and instead highlight the composite functions of government accounts in tourism image construction and public communication.

Specifically, beyond destination promotion, government culture and tourism TikTok accounts also undertake interactive functions such as responding to user inquiries in comment sections, supplementing activity information, and addressing complaints and suggestions. These ongoing interactions constitute an important basis for users’ perceptions of service quality. Accordingly, the concept of “service quality” in this study differs from transaction-oriented service quality in commercial contexts and instead emphasizes dimensions of public communication, responsiveness, and trust-building.

Furthermore, although government accounts may exhibit some similarities to user-generated promotional content in terms of presentation contents, the former offer a more direct perception of destination image due to their official status. Based on these differences in role positioning and functional attributes, the revised manuscript further clarifies the theoretical necessity of focusing specifically on government culture and tourism TikTok accounts.

4. We have revised the Introduction to strengthen the coherence between these sections. Specifically, the revised second paragraph now provides a more structured review of the existing literature and explicitly identifies its limitations. For example, although the tourism literature has examined information quality in social media, it often fails to account for the source of such content. Moreover, the e-government literature has developed robust frameworks for service quality (e.g., reliability, efficiency, responsiveness) but has not extended these to the domain of government social media tourism marketing. This study bridges these gaps by integrating both informational and service-related stimuli within a unified framework.

Building upon this discussion, the third paragraph proposed the present theoretical framework by integrating the SOR model with the emotion appraisal theory to address the gaps in existing research.

5. We have revised the third paragraph of the Introduction to further clarify the rationale for adopting the SOR framework and the emotion appraisal theory, highlighting their suitability and complementary roles in this study. Specifically, the SOR framework provides an overarching analytical structure that explains how external stimuli—namely information quality and perceived service quality in government tourism short videos—relate to travel intentions through individuals’ internal psychological states. However, while the SOR model outlines the stimulus–organism–response process, it offers limited explanation of the internal formation mechanisms within the organism level. To address this limitation, the emotion appraisal theory is incorporated to explain how emotional responses arise from cognitive evaluations, thereby clarifying the internal mechanism linking cognitive perceptions and emotional experiences. This theoretical integration enables a more refined understanding of the dynamic relationship between cognitive and emotional organism states. Based on this integrated perspective, the study proposes that information quality and service quality in government culture and tourism TikTok accounts are not only statistically associated with travel intentions directly, but also indirectly through destination image perception and positive emotional experiences. Furthermore, destination image perception, as a cognitive appraisal outcome, may predict positive emotions, forming a sequential mediating pathway. Through this theoretical integration, the study aims to explain the influence mechanism of government social media tourism communication from a cognition–emotion dual-stage perspective.
6. At the end of the Introduction, we added a concise statement highlighting the theoretical contributions of this study in three aspects. First, we extend the conceptualization of stimulus variables in the context of government social media tourism marketing by incorporating service quality as a relational factor into the analytical framework. Second, we systematically explain how information quality and service quality influence user behavioral responses through distinct relational pathways, thereby constructing a more comprehensive dual-driving mechanism. Third, at the organism level, we further refine the process logic of cognitive appraisal and emotional processing, which contributes to a deeper understanding of the internal psychological mechanisms through which stimuli are transformed into behavioral responses. In addition, in the managerial implications section, the study highlights an operational approach that focuses on enhancing information quality, integrating intelligent service and interaction functions, and following a framework that progresses from cognitive construction to emotional reinforcement. These suggestions are intended to offer useful insights for government tourism social media marketing.

**Comments on the the literature review and research hypotheses**

1. The Literature Review section is currently too descriptive and lacks critical depth. Rather than merely cataloging previous studies, the authors should engage in a more analytical synthesis of the existing body of knowledge.
2. The exposition of emotional appraisal theory is currently inadequate. The manuscript fails to provide a clear, authoritative definition of the theory and lacks a comprehensive review of its recent research progress or evolutionary trajectory within the domain. To strengthen the theoretical framework, the authors should explicitly define the core tenets of the theory and synthesize relevant contemporary studies to demonstrate its current standing and applicability to this research.
3. The integration of the S-O-R framework and emotional appraisal theory within the hypothesis development section remains ambiguous. Specifically, it is unclear how these theoretical lenses are operationalized to deduce the proposed relationships.

**Response:**

1. We have substantially revised the Literature review section to move beyond a descriptive summary of prior studies and to provide a more critical and analytical synthesis of the existing literature. Specifically, rather than merely cataloging previous findings, the revised section critically evaluates the limitations and research gaps within the current body of knowledge. For example, in the discussion of information quality, our review indicates that prior studies have predominantly focused on tourism enterprises, key opinion leaders, and user-generated content, examining how information quality is associated with tourist decision-making, while relatively little attention has been paid to government culture and tourism social media accounts. Regarding service quality, we further identify that existing research mainly examines service quality either within commercial transaction contexts or within e-government service platforms. Limited attention has been given to government social media accounts that simultaneously perform destination image communication and public engagement functions. As a result, existing frameworks cannot fully explain the role of perceived service quality in government tourism short video contexts. Based on this critical synthesis, the revised literature review explicitly identifies the research gap and provides a clearer theoretical foundation for the development of the hypotheses, thereby strengthening the logical connection between prior research and this proposed model.
2. We have revised the Introduction to provide a clearer and more comprehensive theoretical foundation. Specifically, the revised manuscript now includes a more explicit definition of emotional appraisal theory, clarifying its central premise that emotions arise from individuals’ cognitive evaluations of environmental stimuli. In addition, we reviewed relevant studies to demonstrate that emotional appraisal theory has evolved into a key theoretical framework for explaining the relationship between cognitive processing and emotional responses. Building on this foundation, the present study integrates the emotion appraisal theory with the SOR framework to develop an analytical pathway linking stimulus, cognitive appraisal, emotional responses, and travel intentions, thereby strengthening the theoretical foundation of the proposed research model.
3. We acknowledge that the integration between the SOR framework and the emotion appraisal theory was not sufficiently explicit in the previous version of the manuscript. In response to this suggestion, we have revised the Introduction and hypothesis development sections to clarify how these two theoretical perspectives are conceptually and analytically integrated. Specifically, we now explicitly explain that the SOR framework provides the overarching logical structure of the study by conceptualizing the process as a sequence of Stimulus–Organism–Response, while the emotion appraisal theory is employed to elucidate the cognitive mechanisms underlying emotional formation within the organism stage.

Building on this integration, the revised manuscript now clearly operationalized the theoretical logic as follows: information quality and service quality are conceptualized as external stimuli that are associated with audience internal psychological states, represented by destination image perception and positive emotions. Drawing on the emotion appraisal theory, we further clarify that emotion responses are linked to prior cognitive evaluations, thereby justifying the sequential relationship between destination image perception (cognitive appraisal) and positive emotions (emotion response). These internal states subsequently predict audience travel intentions. Accordingly, we have refined the theoretical explanation supporting the proposed sequential mediation pathway of “stimulus → destination image perception → positive emotions → travel intention.” These revisions aim to make the theoretical derivation of the hypotheses more transparent and logically grounded.

**Comments on the the research design**

1. It is crucial to understand how the concept of 'government culture and tourism Douyin accounts' was introduced to the study participants. The manuscript should explicitly detail the instructions, definitions, or visual examples provided to respondents to ensure they correctly identified the specific type of account under investigation. The authors need to clarify this procedure in the methodology section to rule out potential confusion with general travel influencers or commercial accounts.
2. The reporting of sample characteristics regarding usage duration is currently incomplete. While it is noted that 66% of respondents spend 30 minutes to 2 hours daily, the distribution of the remaining 34% is not disclosed. Given the study's focus on Douyin, were specific screening criteria applied to ensure all respondents are active users? The authors must clarify the usage patterns of the remaining participants and justify whether the entire sample possesses sufficient platform experience to provide valid responses.

**Response:**

1. We fully agree that it is essential for respondents to clearly understand the concept of government culture and tourism TikTok accounts to avoid confusion with general travel influencers or commercial marketing accounts. To address this issue, a standardized explanation of the concept was included on the first page of the questionnaire at the outset of the survey. Specifically, respondents were informed that such accounts are officially operated and certified by culture and tourism administrative departments at various governmental levels or their affiliated public institutions. These accounts typically carry official platform verification badges, primarily disseminate destination promotion content. Following your suggestion, we have now explicitly incorporated this clarification into the sample collection procedure.
2. To ensure that all participants had sufficient familiarity with the research context, this study adopted a screening criterion during data collection. Specifically, respondents were required to confirm whether they had previously watched short videos posted by government culture and tourism TikTok accounts. Only participants who reported actual exposure to such content were allowed to proceed with the questionnaire, thereby ensuring that all respondents possessed direct experience with the research object. In addition, the questionnaire collected information on respondents’ average daily TikTok usage duration. The complete distribution is as follows: 66% of respondents reported using TikTok for 30 minutes to 2 hours per day, 8.9% used the platform for more than 2 hours daily, and 25.1% reported less than 30 minutes of daily usage. Overall, the sample is dominated by medium- to high-frequency users. This indicates that most respondents have relatively stable and continuous experience with the TikTok platform, including familiarity with its content ecosystem, interaction mechanisms, and information presentation formats. Therefore, participants were well positioned to provide reliable perceptual evaluations of the information and service performance of government culture and tourism TikTok accounts.

**Comment on the conclusions and implications**

1. The Discussion section currently functions more as a summary of the results rather than a platform for theoretical synthesis. The authors predominantly reiterate their findings without explicitly articulating the study's theoretical contributions. Crucially, there is a lack of critical dialogue with the existing literature; the authors need to discuss how their findings either corroborate, contradict, or extend previous studies to clearly delineate the study's unique value to the field.

**Response:**

1. Following your suggestion, we carefully re-examined the structure and analytical depth of the Discussion and have substantially rewritten this section to strengthen theoretical integration and literature dialogue. The main revisions are summarized as follows:

First, we have introduced the heuristic–systematic model (HSM) as a complementary theoretical perspective to interpret the finding that information quality demonstrates a partial mediation effect, whereas service quality exhibits a full mediation effect. By distinguishing between heuristic processing triggered by high source credibility and systematic processing required for evaluating service-related interactions, we provide a cognitive-processing explanation for the structural differences in how the two types of stimuli are associated with behavioral intention. This revision elevates the discussion from a descriptive interpretation of results to a theory explanation of underlying mechanisms.

Second, our findings confirm that destination image perception and positive emotions mediate the relationships between information quality, service quality, and audience travel intentions. This result corroborates prior studies emphasizing the mediating roles of cognitive and emotional responses in tourism decision-making. By validating this mechanism within the specific context of government culture and tourism TikTok accounts, the study extends existing research into the domain of public-sector social media tourism marketing.

Third, we explicitly reconnect the empirical findings to the theoretical frameworks of the SOR model and the emotion appraisal theory. In particular, regarding the internal structure of the organism stage, our results reveal that destination image perception precedes and predicts positive emotions. This finding differs from some previous studies proposing an emotion-first pathway. Drawing on the emotion appraisal theory, which posits that emotions arise from prior cognitive evaluations, we provide a theoretical explanation for this sequence, thereby refining the internal explanatory logic of the SOR framework within a government social media tourism marketing context.

**Comment on the limitations and future research**

1. Several of the acknowledged limitations are remediable within the current research scope.

**Response:**

1. Following your suggestion, we have substantially revised the Limitations section. The revisions mainly include the following aspects:

First, we explicitly acknowledge the limitations of the cross-sectional research design in supporting causal inference and suggest that future studies adopt longitudinal or experimental approaches to better establish causal relationships among variables.

Second, we emphasize the context-dependent nature of this study, which is situated within government culture and tourism TikTok accounts and shaped by platform characteristics. Accordingly, the external validity of the findings requires further examination across different platforms and cultural contexts.

Third, we discuss the limitations of the model, particularly its reliance on linear structural assumptions. To better capture the complexity of user responses in social media environments, future research should explore potential nonlinear relationships and dynamic mechanisms.

We sincerely appreciate your valuable feedback. Through these revisions, we have strengthened the suitability and integration of the theoretical framework, and expanded key methodological and theoretical discussions. We hope that the revisions have adequately addressed your concerns and substantially improved the manuscript’s theoretical rigor and readability.

**Response to Reviewer 4:**

**Comment：**

Reviewer #4: Dear Authors,
Thank you for submitting your work to PLOS One. I have had the opportunity to revise your paper, “Investigating the Effects of Information Quality and Service Quality of Government Culture and Tourism Douyin accounts on Audience Travel Behavioral Intentions.” This manuscript represents a methodologically sound and theoretically grounded contribution to the literature on government social media, tourism communication, and short-video platforms. I find it generally well written and potentially interesting to readers.
The manuscript is generally clear, well-structured, and intelligible, and the argument progresses logically from theory to hypotheses, methods, results, and implications. The literature review is comprehensive, and the discussion appropriately links findings back to theory and practice. The manuscript would further benefit from language polishing and copyediting (e.g., occasional redundancy and overly long sentences, particularly in the Introduction and Conclusions; grammatical errors and phrasing; inconsistent use of terminology such as “image perceived” vs. “image perception”; minor formatting and spacing issues in tables and hypothesis labeling). These issues do not undermine the scientific content but should be addressed to improve clarity and readability.
Overall, the manuscript presents a technically sound empirical study grounded in well-established theoretical frameworks (SOR model and emotion appraisal theory). The research questions are clearly articulated, the conceptual model is logically derived from literature, and the hypothesized relationships are theoretically justified. The study relies on survey data from 406 respondents, which is an adequate sample size for SEM. Measurement scales are adapted from prior validated studies, and the authors follow appropriate procedures for scale adaptation, including translation/back-translation and expert review. Reliability and validity assessments meet commonly accepted thresholds, supporting the adequacy of the measurement model. The SEM results are coherent and largely consistent with the proposed hypotheses. The distinction between the effects of information quality (direct and indirect) and service quality (fully mediated) is empirically supported and interpreted in a theoretically meaningful way. The conclusions regarding the “content-first” logic of government Douyin accounts are reasonable given the data. The outcome variable is behavioral intention rather than actual behavior, a limitation the authors appropriately note. The only theoretical issue worth improving is, in my opinion, a potential redundancy and overlap between information quality, destination image perception and positive emotions. For example, high-quality tourism videos by definition shape destination image, while emotional responses are tightly embedded in image formation. While discriminant validity statistics pass, this does not fully resolve conceptual overlap (information quality predicts image, which predicts intention, but image is partly constructed through information quality). Perhaps you could clarify temporal or cognitive ordering? (e.g., cognition --> affect --> image --> intention). Overall, the conclusions are appropriately drawn from the data and do not overreach excessively the empirical evidence, but the findings should be interpreted within the acknowledged methodological constraints (i.e., non-probability sampling, cross-sectional design, self-reported measures). That said, I would like to call the attention tosomewhat overconfident causal interpretation and suggest reframing conclusions using associational language (using e.g., expressions such as "is associated with”, “predicts”, or “is linked to”) and acknowledging causal limitations more explicitly.
Statistical analysis is generally rigorous and appropriate for the research objectives. However, I believe the manuscript would benefit from a clearer explanation of how higher-order constructs (e.g., information quality and service quality) were modeled, that is, whether as second-order factors or aggregated dimensions. Also, some hypotheses (e.g., H3) appear conceptually redundant or mislabeled, which may reflect minor inconsistencies in hypothesis numbering rather than analytical flaws. Despite these minor issues, the analytical strategy is sound, competently executed, and aligned with current standards in tourism and social media research
The authors state that all relevant data are within the manuscript and its supporting information files. This is formally compliant with the PLOS Data Policy. However, based on the manuscript text alone, it is not entirely clear whether item-level response data (i.e., the data underlying means, variances, and SEM analyses) are fully accessible. If privacy or ethical constraints apply, these should be stated explicitly. Clarifying this point would strengthen compliance with PLOS ONE’s open data requirements.

**Response：**

We sincerely thank you for your positive evaluation of our manuscript and for your thoughtful and constructive comments. We greatly appreciate your recognition of the study’s theoretical grounding and methodological rigor. Following your suggestions, we have carefully revised the manuscript to improve clarity, theoretical precision, and transparency. Our responses and revisions are summarized below.

1. Language polishing and terminology consistency. Following your suggestion, we conducted a comprehensive revision of the manuscript to improve language quality and readability. We focused on enhancing grammatical accuracy, clarity of expression, and academic writing standards. Throughout the paper, we corrected grammatical errors, awkward phrasing, and “Chinglish” expressions, shortened overly long sentences, removed redundant wording, and standardized terminology usage (e.g., consistently using “image perception”). Additionally, we conducted a thorough review of the entire manuscript. We revised potentially causal expressions such as “affect,” “influence,” “drive,” and “enhance,” replacing them with more cautious, association-based wording, such as “is positively associated with,” “is related to,” “is linked to,” and “predicts.” For example, all instances of "influence" in the hypotheses have been revised to "is positively associated with." Moreover, formatting was adjusted to ensure full compliance with PLOS ONE requirements.
2. We appreciate your insightful observation regarding the potential conceptual overlap among information quality, destination image perception, and positive emotions. Following your suggestion, we further clarified the theoretical logic underlying the relationships among these constructs. Drawing upon the SOR framework and the emotion appraisal theory, we explicitly articulate a sequential cognitive–emotional process in the revised manuscript. Specifically, external stimuli (information quality and service quality) first are associated with destination image perception as a cognitive evaluation, which subsequently predicts positive emotional responses, ultimately leading to travel intentions. This clarification emphasizes the temporal and cognitive ordering of constructs and reinforces the conceptual distinction between cognition-based evaluation and emotional reactions. The revised theoretical explanation has been incorporated into the Introduction and Hypothesis sections.

To assess the discriminant validity of the constructs, we added a confirmatory factor analysis in the data analysis section. We compared the baseline measurement model with alternative models. The results show that the latent variables of this study are clearly distinct.

3. Clarification of higher-order construct modeling. To improve methodological transparency, we added a clearer explanation of how higher-order constructs were modeled. Information quality and service quality are now explicitly described as second-order constructs.
4. Optimization of hypothesis. Following your suggestion and drawing on the SOR model and emotion appraisal theory, we proposed hypotheses on the sequential mediating role of cognitive ordering and tested them. Redundant or unnecessary hypotheses were removed, resulting in a clearer framework of eight hypotheses, including direct effects, parallel mediation, and sequential mediation.
5. Data availability clarification. Thank you for pointing out the need for clearer compliance with PLOS ONE’s Data Policy. Survey questionnaire and dataset used for analysis are provided in the supporting information files All data have been fully anonymized to protect participant privacy, and no ethical restrictions limit data sharing.

We sincerely appreciate your constructive and encouraging feedback. Your insightful comments have helped us substantially improve the clarity and theoretical rigor of the manuscript.
