# Supplementary material for: Understanding travel intention formation in government culture and tourism TikTok accounts: An integration of the SOR model and emotion appraisal theory
Source: PLoS One. 2026 May 18;21(5):e0349305. doi: 10.1371/journal.pone.0349305 (PMC13183215; doi:10.1371/journal.pone.0349305)
Supplement: Attachment — Submitted filename: Minor Revision.docx [file pone.0349305.s005.docx]

# Reviewer Report

# General Evaluation

This manuscript investigates the formation of audience travel intentions in the context of government culture and tourism TikTok accounts (GCTTAs) by integrating the Stimulus–Organism–Response (SOR) model with emotion appraisal theory. The study examines the roles of information quality and service quality, as well as the mediating effects of destination image perception and positive emotions, using survey data and structural equation modeling.

Overall, the topic is timely and relevant, especially given the growing importance of government social media in destination marketing. The manuscript demonstrates good potential for publication. The theoretical framing is clearer, the methodological procedures are reasonably transparent, and the empirical findings are meaningful.

However, several issues still need to be addressed to further strengthen its theoretical contribution, conceptual clarity, and overall rigor.

# Major Comments

**1. Theoretical contribution needs further sharpening**

The manuscript would benefit from a more explicit articulation of its theoretical contributions beyond applying the SOR model in a new context. The authors should clarify how service quality in government social media differs from traditional commercial contexts, and how the finding of full mediation contributes to existing theory.

**2. Conceptual distinctiveness between information quality and service quality**

Although discriminant validity is supported statistically, some measurement items may conceptually overlap with platform or content characteristics. Further clarification is needed regarding why these items are categorized as service quality.

**3. Interpretation of the non-significant direct effect of service quality**

The finding that service quality has no direct effect but only indirect effects is important. The discussion could be strengthened by exploring deeper theoretical explanations, such as trust-building mechanisms and differences between short-term and long-term effects.

**4. Robustness checks could be further strengthened**

The manuscript includes some robustness tests, but additional analyses (e.g., alternative model specifications or subgroup analysis) would further enhance confidence in the results.

**5. Language and academic writing quality**

Minor grammatical issues and awkward phrasing remain. A final round of professional English editing is recommended.

# Minor Comments

1. Ensure consistency in terminology across the manuscript.

2. Maintain consistent use of non-causal language.

3. Clarify the specific platform context (Douyin vs TikTok).

4. Improve table formatting and consistency.

# Recommendation

Minor Revision

The manuscript is well-developed and has good potential for publication. With further refinement, it would be suitable for acceptance.
