# Supplementary material for: Understanding travel intention formation in government culture and tourism TikTok accounts: An integration of the SOR model and emotion appraisal theory
Source: PLoS One. 2026 May 18;21(5):e0349305. doi: 10.1371/journal.pone.0349305 (PMC13183215; doi:10.1371/journal.pone.0349305)
Supplement: Attachment — Submitted filename: Response_to_Reviewers_auresp_2.doc [file pone.0349305.s007.doc]

Response Letter

Dear Editor and Reviewers,

We would like to sincerely thank you for your constructive and insightful comments on our manuscript entitled "Understanding travel intention formation in government culture and tourism TikTok accounts: An integration of the SOR model and emotion appraisal theory" (ID:PONE-D-25-66483R1). We greatly appreciate the time and effort you have devoted to reviewing our work. Your suggestions have been invaluable in helping us improve the clarity, rigor, and theoretical contribution of the manuscript.

We have carefully revised the manuscript to ensure that all changes are clearly presented, and we hope that the revisions adequately address your comments. The main revisions are summarized as follows:

1. Redundant expressions have been removed throughout the manuscript to improve conciseness and clarity.
2. Typographical errors, grammatical issues, and awkward phrasing have been revised to enhance readability and academic rigor.
3. Terminology has been standardized across the manuscript. For example, terms such as “government departments” and “public sector” have been consistently revised to “government culture and tourism departments” to ensure conceptual consistency.
4. The definition of service quality has been revised to establish a clearer conceptual distinction from information quality.
5. The theoretical contributions have been further refined and strengthened. In particular, the full mediation effect of service quality has been elaborated, highlighting its context-dependent nature, whereby its role is jointly shaped by the characteristics of information sources, communication objectives, and social media platform features.
6. Additional robustness checks have been conducted using subgroup analysis to further enhance the reliability of the findings.
7. The discussion of the non-significant direct association between service quality and travel intention has been further developed. Following the reviewers’ suggestions, alternative explanations have been incorporated, including the role of trust development, differences between short-term and long-term effects, and the characteristics of short-video consumption contexts.

We believe that these revisions have improved the overall quality and rigor of the manuscript. We sincerely appreciate the time and constructive feedback provided by the editor and reviewers, and we look forward to your further evaluation.

Sincerely,

Yuan Sun (on behalf of all co-authors)

**Response to Reviewer 1:**

**Comment1：**

Reviewer #1: This manuscript addresses a timely and meaningful topic by examining how government culture and tourism TikTok accounts shape audience travel intentions. The study is well positioned at the intersection of tourism marketing, government social media communication, and short-video platform research, and it offers a clear attempt to extend the SOR framework through the integration of emotion appraisal theory. The paper is generally well organized, the theoretical framing is more focused than in many platform-based behavioral studies, and the empirical analysis is sufficiently solid to support the main claims. In particular, the distinction between information quality and service quality, as well as the identification of destination image perception and positive emotions as both parallel and sequential mediators, gives the paper a meaningful analytical structure. Overall, I find the manuscript publishable in its current form and recommend acceptance.

1. The topic selection is meaningful and has both theoretical and practical relevance. Rather than examining generic tourism short-video content or commercial accounts, the manuscript focuses specifically on government-operated culture and tourism TikTok accounts, which gives the study a clearer institutional context and a stronger public communication dimension. This is important because such accounts differ from ordinary commercial or user-generated content in that they combine destination promotion with public communication and online responsiveness. The paper therefore engages with a research object that is not only contemporary and policy-relevant, but also theoretically capable of generating new insights beyond mainstream tourism social media studies.

2. The language of the manuscript is generally fluent and appropriate for an international academic journal. The revised version reads much more professionally than a typical platform-behavior paper, and most sections are expressed in clear academic English with a relatively stable tone. Key constructs are defined in a readable way, the transitions between theory, hypotheses, methods, and results are mostly smooth, and the discussion section in particular shows improved precision in wording by avoiding overly strong causal claims in a cross-sectional design. This makes the manuscript easier to follow and improves its overall scholarly presentation.

3. The innovation and argumentation are clearly articulated. The paper does more than simply “apply SOR in a new setting.” Its main contribution lies in showing that information quality and service quality operate through different relational pathways in the context of government tourism social media. The finding that information quality has both direct and indirect associations with travel intention, whereas service quality operates through full mediation, is theoretically interesting and is explained in a reasonably convincing way through the added heuristic–systematic processing perspective. In addition, the manuscript refines the organism component by distinguishing cognitive appraisal from emotional response and by empirically supporting a cognition-to-emotion progression. This gives the theoretical model a stronger internal logic and helps the paper stand out from many studies that treat mediators in a more generic or interchangeable manner.

4. The article provides sufficiently developed discussion and does not stop at merely reporting statistical significance. The revised discussion links the main findings back to the SOR framework and emotion appraisal theory, and it also attempts to explain why service quality functions differently in a government communication context than it might in a commercial setting. I also appreciate that the authors compare their findings with prior studies, discuss the ordering between destination image perception and positive emotions, and derive managerial implications that are aligned with the empirical results rather than being overly generic. The move from cognitive construction to emotional reinforcement is especially well connected to the reported mediation structure.

5. The manuscript is methodologically and formally in ways that support confidence in the findings. The authors provide details on questionnaire adaptation, sampling procedures, recruitment channels, duplicate-response controls, and response-time screening. They report reliability and validity indicators, compare competing CFA models, add SRMR, and supplement Harman’s single-factor test with an unmeasured latent method factor approach. The reporting of sample characteristics, measurement items, ethics statement, conflict-of-interest statement, funding information, and data availability is also relatively complete. These features give the paper a level of formal rigor and transparency that is appropriate for publication.

**Response：**

We sincerely thank you for the positive and encouraging evaluation of our manuscript. We greatly appreciate your recognition of the study’s theoretical contributions, methodological rigor, and clarity of presentation. At the same time, we have carefully proofread the entire manuscript to further improve its overall quality and clarity of expression.

Thank you again for your valuable and constructive comments, the time you have devoted, and your support for our study.

**Response to Reviewer 2:**

**Comment1：**

The manuscript investigates the formation of audience travel intentions in the context of government culture and tourism TikTok accounts (GCTTAs) by integrating the Stimulus–Organism–Response (SOR) model with emotion appraisal theory. The topic is timely and relevant, particularly given the increasing role of government social media in destination marketing and public communication.

Overall, the manuscript is well-structured, and the research design is generally sound. The integration of information quality and service quality within a unified analytical framework, along with the examination of destination image perception and positive emotions as mediators, provides a meaningful contribution to understanding behavioral mechanisms in this context. The empirical analysis is clearly presented, and the results are coherent and interpretable.

However, several issues should be addressed to further strengthen the manuscript:

First, the theoretical contribution could be further sharpened. While the manuscript highlights its contributions more clearly than before, part of the contribution still appears to be framed as applying existing theories to a new context. The authors are encouraged to more explicitly articulate how the findings—particularly the fully mediated role of service quality—extend or refine existing theoretical perspectives, especially in relation to service quality and public-sector communication.

**Response1：**

We sincerely appreciate you for the insightful and constructive comments. Your suggestions provided valuable guidance for refining the theoretical positioning and rigor of the manuscript, and we have carefully revised the relevant contents accordingly.

We agree that merely applying existing theories to a new context is insufficient to establish a strong theoretical contribution. Following your suggestion, we have further strengthened the theoretical contribution of this study.

Specifically, our findings indicate that, in the context of social media marketing by government culture and tourism departments, service quality primarily operates through cognition–emotion pathways, rather than exerting a direct influence on audiences’ travel intentions. This fully mediated effect challenges the commonly held assumption in prior research that service quality directly affects behavioral outcomes. Furthermore, we emphasize that the role of service quality is contingent upon multiple factors, including the nature of the information source, communication objectives, and the characteristics of short-video platforms. This study provides a more nuanced understanding of the mechanisms through which service quality operates in public-sector tourism communication.

**Comment2：**

Second, although the manuscript distinguishes between information quality and service quality conceptually, some measurement items related to service quality may overlap with content or platform characteristics. Additional clarification would help strengthen the conceptual distinctiveness between these constructs and improve construct validity.

**Response2：**

Thank you for this insightful and important comment. We agree that clearly distinguishing service quality from information quality is essential for improving construct validity.

Following your suggestion, we have further strengthened the conceptual distinction between these two constructs in the manuscript. Specifically, we clarify that information quality refers to the evaluation of content attributes of short videos, whereas service quality is conceptualized as an account-level construct that captures audiences’ perceptions of the overall performance of government tourism departments in delivering information and services via the TikTok platform.

In addition, we have refined the definitions of the three dimensions of service quality (reliability, responsiveness, and assurance), emphasizing their interaction-oriented and service-oriented nature rather than content-related attributes, thereby avoiding potential conceptual overlap with information quality.

We hope that the above revisions help establish a clearer conceptual boundary between the two constructs and further enhance construct validity.

**Comment3：**

Third, the discussion of the non-significant direct effect of service quality on travel intentions could be further deepened. This finding is potentially important, and the authors may consider elaborating on alternative explanations, such as the role of trust-building, differences between immediate and long-term effects, or characteristics of short-video consumption environments.

**Response3：**

Following your suggestion, we have further expanded the discussion of this finding from multiple perspectives.

First, from the evaluative nature of service quality, we note that such evaluations rely on continuous interaction and feedback, making them difficult to form through cognitive shortcuts in a single exposure. Based on the Heuristic–Systematic Model (HSM), service quality is therefore more likely to operate through the systematic processing route rather than serving as an immediate heuristic cue to influence behavioral intentions.

Second, we introduce a temporal perspective by distinguishing between short-term and long-term effects, emphasizing that the influence of service quality unfolds through an accumulation process.

Third, considering the characteristics of short-video consumption environments, we point out that algorithm-driven recommendation mechanisms and fast-paced content exposure reinforce users’ reliance on heuristic processing. This, in turn, constrains the immediate impact of service quality, which depends on systematic evaluation.

**Comment4：**

Fourth, while the manuscript includes robustness checks such as competing model comparisons and common method bias tests, additional robustness analyses (e.g., alternative model specifications or subgroup analysis) would further enhance confidence in the findings.

**Response4：**

Following your suggestion, we have conducted additional robustness analyses by implementing multi-group structural equation modeling (MGSEM) to examine the stability of the proposed model across different subgroups. Specifically, we performed multi-group invariance tests based on gender, age, and usage frequency.

Following established procedures, we assessed configural, metric, and structural invariance by comparing a sequence of nested models with increasingly restrictive parameter constraints. The results (Table 4) indicate that the unconstrained baseline models demonstrate acceptable fit across all groups. As constraints were progressively imposed, the changes in model fit indices (e.g., ∆CFI) remained minimal and well within recommended thresholds.

Although the chi-square difference test is statistically significant in a few cases, the corresponding ∆CFI values remain below the critical value of 0.01, suggesting that the deterioration in model fit is negligible and that the invariance assumption is not violated.

Overall, the findings from the multi-group analyses demonstrate that the measurement and structural relationships are stable across gender, age, and usage frequency groups. This provides additional evidence for the robustness of the proposed model and supports the generalizability of the results across different demographic and behavioral segments.

**Comment5：**

Fifth, regarding data availability, the manuscript states that all relevant data are provided within the manuscript and its Supporting Information files, which is consistent with the journal’s policy. The authors are encouraged to ensure that the dataset is clearly documented (e.g., variable definitions and coding schemes) to facilitate reproducibility.

**Response5：**

The questionnaire instrument and the complete dataset have already been provided in the manuscript and the Supporting Information files. The dataset is based directly on the original measurement scales, and all variables correspond closely to the questionnaire items. Therefore, the data structure is transparent and straightforward, enabling straightforward interpretation and reproducibility without the need for additional coding schemes.

In addition, we have carefully reviewed the dataset and the corresponding descriptions in the manuscript to ensure consistency and clarity, so that the analysis can be easily reproduced.

We hope that the current level of documentation is sufficient to support transparency and reproducibility.

**Comment6：**

Finally, the manuscript is generally understandable, but minor grammatical errors and awkward phrasing remain. A final round of professional English editing is recommended to improve clarity and readability.

In summary, the manuscript has good potential for publication. With the above revisions, it would be suitable for acceptance.

**Response6：**
We have carefully revised the manuscript to address minor grammatical errors, punctuation issues, redundant expressions, and awkward phrasing, in order to ensure that the manuscript meets the standards of academic English. We hope that these revisions have improved the overall quality and readability of the manuscript.

Once again, we would like to express our sincere gratitude for your careful review and insightful comments.
